# How Does *Barbe-Bleue* Subjugate His Wives? Psychological and Social Coercion of Women in Interpersonal Power Contexts: A Scoping Review

**DOI:** 10.3390/bs16060983

**Published:** 2026-06-12

**Authors:** Elena Duque-Sánchez, Tinka Schubert, Carme Garcia-Yeste, Oriol Rios

**Affiliations:** 1 Department of Theory and History of Education, University of Barcelona, Pg Vall d’Hebron, 171, 08035 Barcelona, Spain; 2 Faculty of Political and Social Sciences, Universität der Bundeswehr München, Werner-Heisenberg-Weg 39, 85577 Neubiberg, Germany; tinka.schubert@unibw.de; 3 Department of Pedagogy, Rovira I Virgili University, 43007 Tarragona, Spain; carme.garciay@urv.cat (C.G.-Y.); joseoriol.rios@urv.cat (O.R.)

**Keywords:** subjugation of women, violence against women, psychological coercion, social coercion, gender socialisation, sexual scripts

## Abstract

In Paul Dukas’s opera, *Ariane et Barbe-Bleue*, when the captive wives are allowed to leave their confinement, they refuse to do so and remain with their aggressor. This narrative raises a central question: why do some women remain in violent contexts even when apparent pathways for escape exist? This scoping review aims at analysing the psychological and social mechanisms of coercion employed by those who perpetrate violence, clearly stating the focus on the responsibility of the perpetrator and exploring diverse relationship settings. A total of 31 articles from diverse disciplines such as social psychology, sociology, education, and studies on coercive control have been examined to provide insight into: (1) the psychological and (2) social coercion mechanisms and (3) the influence of gender socialisation on perpetuating the subjugation of women. These mechanisms are analysed transversally across intimate partner relationships and coercive family and community systems. Findings reveal that across these geographically, culturally, and religiously diverse settings, as well as across the diverse relationships analysed, similar patterns of psychological and social coercion exist that are framed and reinforced by a gendered socialisation rooted in patriarchal gender roles.

## 1. Introduction

The figure of *Barbe-Bleue* has been recurrently used as a metaphor for male violence and female submission (Bosna, 2024; Jesussek, 2023; Watson, 2018). There are different versions of the tale, but in Paul Dukas’s opera, *Ariane et Barbe-Bleue*, when Ariane finds the captive wives, and they are allowed to leave their confinement, they refuse to do so and remain with their aggressor. The following fragment of the opera reflects this idea.
**SÉLYSETTE** *(S’élançant après elle et l’arrêtant)* Ariane!… Ariane!… Où vas-tu? **ARIANE** Loin d’ici; là bas, où l’on m’attend encore… M’accompagnes-tu, Sélysette? **SÉLYSETTE** Quand reviens-tu? **ARIANE** Je ne reviendrai pas… **MÉLISANDE** Ariane!… **ARIANE** M’accompagnes-tu, Mélisande? *(Mélisande regarde tour à tour Barbe-Bleue* *et Ariane, et ne répond point)* **ARIANE** Vois, la porte est ouvert et la campagne est bleue… Ne viens-tu pas, Ygraine? *(Ygraine ne tourne pas la tête)*La lune et les étoiles éclairent toutes les routes. La forêt et la mer nous appellent de loin et l’aurore se penche aux voûtes de l’azur, pour nous montrer un monde inondé d’espérance… Venez-vous, Bellangére? **BELLANGÉRE** *(Sèchement)* Non. **ARIANE** Je m’en irai seule, Alladine? *(A ces mots, Alladine court à Ariane, se jette* *dans ses bras et, parmi des sanglots convulsifs,* *la tient longuement et fiévreusement enlacée)* **ARIANE** *(Se dégageant doucement)* Reste aussi, Alladine… Adieu, soyez heureuses… *(Elle s’éloigne, suivie de la Nourrice. Les femmes* *se regardent, puis regardent Barbe-Bleue qui relève lentement la tête. Un silence)*

This narrative raises a central question: why do some women remain in violent contexts, even when apparent pathways for escape exist? When explanations based exclusively on economic dependence, lack of material resources, the fear of the consequences of ending that relationship, absence of external support are set aside, the scientific literature on psychological coercion, gender socialisation, and interpersonal power dynamics offers key insights into this phenomenon. Yet this has not been systematically compiled to further explore the mechanisms that keep women trapped in abusive relationships. Also, feminist literature raises this question. For example, Hooks (2000a) describes a romantic relationship in her book *All About Love. New Visions and states*: When it became destructive, I found it difficult to leave. I found myself accepting behavior (verbal and physical abuse) that I would not have tolerated in a friendship (p. 136).

To begin with, any analysis of why a woman remains in a violent relationship must take into account the broader framework of a patriarchal society (Lerner, 1986; MacKinnon, 1989). In this regard, the work of Johnson and Leone (2005) is particularly noteworthy, as it distinguishes, firstly, intimate terrorism, characterised by a continuous pattern of control, domination, and fear exerted over the partner through various forms of violence. Secondly, they propose situational couple violence, which arises from everyday conflicts that escalate into aggression, without necessarily involving a dynamic of permanent control. This second type is more difficult to identify because it is often normalised, occurs sporadically, and is frequently not socially recognised as gender-based violence or intimate partner violence. Additionally, key concepts such as learned helplessness (Walker, 1979) help explain the psychological paralysis experienced by abused women. Along the same lines, Stark (2007) explains how violence functions as a system of control that—beyond physical aggression—acts as a mechanism that restricts victims’ freedom. In this regard, she also argues that this control involves processes of isolation, exploitation, frightening or physical harm. Ptacek (2023), particularly in Chapters 6 and 7 of his work, demonstrates how these processes are also steeped in a context that contributes to greater oppression whilst simultaneously rendering violence invisible. He refers to contexts such as economic and cultural factors, established patriarchal norms, among others. Similarly, Herman (2022) analyses how complex trauma alters a person’s capacity to respond to aggression.

When research emphasises why a woman chooses to remain with her aggressor—examining her socialisation, beliefs, preferences, and understanding of relationships—it cannot ignore the key elements mentioned above. Thus, Hooks (2004) argues that this socialisation is shaped by patriarchy, which dictates the most appropriate attitudes for men and women whilst simultaneously perpetuating relationships of male domination. Butler (1990) also argues that socialisation is closely linked to gender performativity, which is entirely defined by norms that perpetuate relationships of power and exclusion. In short, if these factors are overlooked, there is a risk of blaming women for their “decision” and promoting victim blaming (Miao & Westmarland, 2025).

For example, the well-known Stockholm Syndrome is sometimes explained in a reductionist way through statements such as “she fell in love with her kidnapper,” which is a misleading explanation. This syndrome does not imply that the victim feels attraction toward violence or toward the aggressor. Rather, it describes how victims develop psychological defence mechanisms in situations of coercion that distort their perception of reality, leading them to reject those who try to help them while supporting those who harm them. In this context, the victim attempts to reduce cognitive dissonance to minimise psychological distress (Litinetskaia & Guelfi, 2021).

While acknowledging the complexity of sexual violence in terms of affected genders and perpetrating genders, this scoping review focuses on heterosexual relationships and on violence perpetrated by men against women. Specifically, we start from the definition of interpersonal violence as violence within intimate relationships that can cause physical, sexual, or psychological harm (Rosenberg et al., 2006). Furthermore, we also consider symbolic violence as that which perpetuates domination over women through power relations, with the complicity of one or more individuals in maintaining it (McRobbie, 2004). This is meant to operationalise the search requests, as well as to adequately respond to the myth of women remaining in violent relationships due to their attachment. We also explicitly reject any analysis that promotes victim-blaming. Furthermore, the paper is not focused on psychological mechanisms that may prevent women from leaving abusive relationships. Instead, the paper concentrates on the mechanisms of submission imposed on women that lead them to remain in situations of violence. Accordingly, the study focuses on the mechanisms of manipulation, persuasion, and coercion used to subjugate women. Rather than asking “Why didn’t she leave?”, this paper addresses the question “How was she prevented from leaving?”

To this end, the review examines the coercive, controlling and manipulative mechanisms operating across three domains: the abusive individual, the victim’s social environment (friends, family, broader context, etc.), and the gender scripts embedded in the society in which these mechanisms occur. Based on this framework, three research questions are formulated:

1. What are the psychological coercion mechanisms employed by those who perpetrate violence to subjugate and manipulate women?
2. What are the social coercion mechanisms employed by those who perpetrate violence to subjugate and manipulate women?
3. How does gender socialisation influence and/or legitimise the interpersonal and social power dynamics that subjugate women?

## 2. Materials and Methods

To elaborate on the scoping review, we have conducted a systematic review following the PRISMA Extension for Scoping Reviews (PRISMA ScR) guidelines (Tricco et al., 2018), assuring the quality of the analysis and the adequate response to the abovementioned questions.

### 2.1. Eligibility Criteria, Information Sources and Search

To develop the scoping review, the search was conducted in two databases, Web of Science and Scopus. Date restrictions were applied from 1 January 2016 to 20 February 2026, and sources were restricted to scientific journal articles. To collect the maximum information for covering the research questions, we used the following combinations of keywords (see Table 1):

These keywords were applied in four structured search combinations in each database. All searches shared a common base string: (“women” OR “girl” OR “female”) AND (“gender based violence” OR “gender violence” OR “intimate partner violence” OR “sexual violence”). To this base, one additional conceptual component was added in each search: (1) (“coercion” OR “control”), (2) “psychological manipulation”, (3) “traumatic bonding”, or (4) (“gender socialisation” OR “gender scripts”).

Authors agreed to limit the number of retrieved records to a maximum of 150 results per search. In cases where the number of retrieved records exceeded this limit (two combinations in Web of Science and one in Scopus), additional refinement terms were systematically applied (e.g., “manipulation”, “power”, “subjugation” OR “submission”) using the AND operator. These terms were selected because they are conceptually aligned with the focus of the review on coercion, control, and mechanisms of subjugation. This refinement ensured a reduction in the number of records while maintaining conceptual relevance to the research questions. This decision was made to ensure the feasibility and manageability of the screening process within the scope of this scoping review. The authors acknowledge that this decision may introduce selection bias and, therefore, constitutes a limitation of the study. To mitigate this risk, screening procedures were complemented by careful manual review. When results exceeded this number, additional keywords were applied to further refine the results. Finally, 275 records were identified, including 224 from Web of Science and 51 from Scopus.

### 2.2. Selection of Sources of Evidence

All identified records were imported into Excel files. AI-assisted tools (ChatGPT, OpenAI GPT 5.5 thinking) were used to support file merging and the identification of potential duplicates based on bibliographic information. The outputs were systematically verified through manual review by the authors to ensure accuracy and completeness, thereby maintaining full human oversight. A total of 33 records were removed after being identified as duplicates. As a result, 242 records remained.

Then, the four authors began by independently screening all identified records. They selected the records by reading all the titles and abstracts, excluding records that did not address the research questions. Doubts about any record were discussed with the other authors. From this human review, 155 records were removed, and 87 remained.

Then, access to the full texts of all the records was reviewed, ensuring their availability, their scientific nature, and that they were written in one of the following five languages: English, Spanish, German, French and Italian. From this review, one record was removed for not being a scientific article, but a conference proceeding. Three records were removed for being in other languages, and two more records were removed as the full text was not available. The screening resulted in 81 reports.

Then, authors elaborated a list of inclusion and exclusion criteria, located in Table 2, to ensure the reports can help respond to the research questions. The authors employed AI-assisted tools integrated within their institutional research infrastructure (e.g., Microsoft Copilot integrated in Microsoft 365, Microsoft Corporation, GPT-5–based model) to support the identification of elements related to the inclusion and exclusion criteria. These tools were used solely to assist in identifying potentially relevant information. All AI-generated outputs were critically assessed and manually reviewed by the authors to ensure adherence to the predefined criteria, thereby guaranteeing human oversight and methodological reliability. AI tools were not used to make eligibility decisions, conduct final data extraction, or interpret or synthesise the findings. From this screening, 42 reports remained.

### 2.3. Data Charting and Data Items

The 42 reports were entered into a data charting table, including author(s), title, methodology, participants, and the main contributions related to the three research questions. The authors read each report in depth and drafted the relevant sections accordingly.

### 2.4. Synthesis of Results

**Figure 1:**
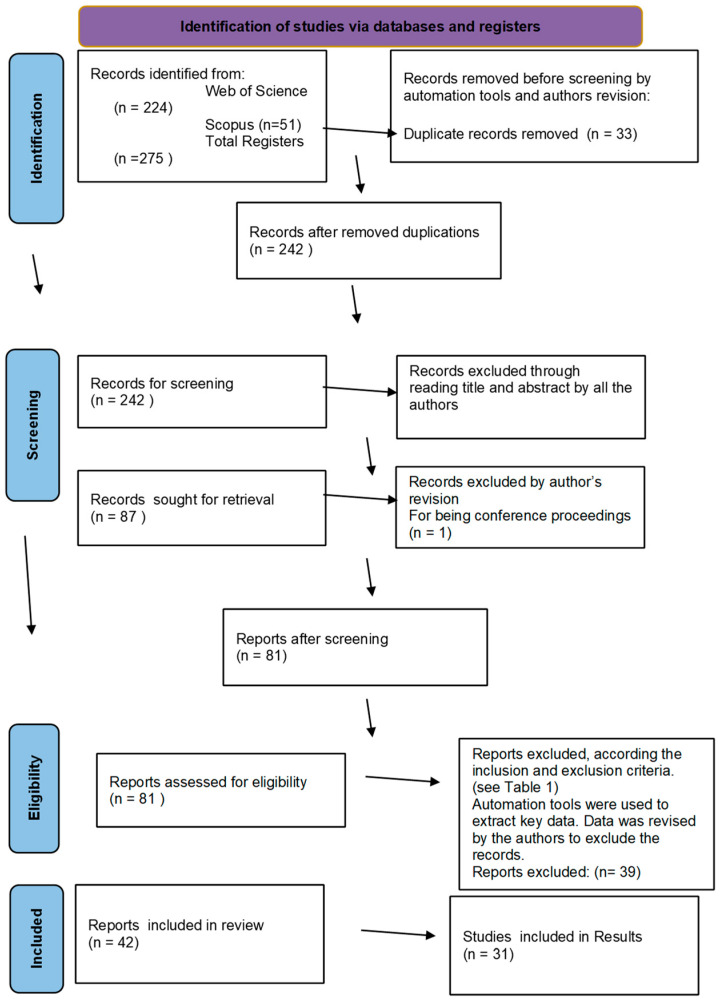
Prisma diagram flow.

We grouped the reports by the three research questions and summarised the main contributions. During this process, the authors identified some articles that did not provide meaningful contributions to the research questions. When a report did not provide information relevant to any of the three questions, it was excluded from the final set of results. Finally, 31 studies remained. The complete study selection process is shown in the PRISMA flow diagram (Figure 1).

## 3. Results

In this section, we present the results of the analysis of the 31 articles ultimately selected to address the three questions regarding how psychological and social coercion is exercised against women in interpersonal power contexts. We organise these results into a first section on the psychological coercion mechanisms employed by those who perpetrate violence to subjugate and manipulate women. A second section on the social coercion mechanisms employed by those who perpetrate violence to subjugate and manipulate women. And finally, a third section on how gender socialisation influences and/or legitimises the interpersonal and social power dynamics that subjugate women.

First, Table 3 presents a summary of the findings from the 31 studies ultimately included in the review.

### 3.1. Mechanisms Employed by Those Who Perpetrate Violence

A total of 13 of the 31 final articles selected explicitly discuss the mechanisms used by men who employ psychological coercion to subjugate and manipulate women.

As Schneider (2018) notes, coercive control is difficult to identify as violence against a partner or against women. In many cases, there is no physical violence, but there is a constant fear of upsetting the abuser. Fear that they will shout at you, humiliate you, blackmail you, bully you, ignore you, or otherwise jeopardise your freedoms (p. 49). This has an effect on the victim, who constantly monitors their behaviour towards the perpetrator to minimise the harm they suffer. Furthermore, as Zapcic et al. (2024) point out, there is no cultural, personal, or geographical profile of a victim of psychological coercion, meaning that any woman could be a potential victim.

The identification of this type of violence, which is so difficult for women themselves—and even for society—to detect, should, in Schneider’s (2018) view, lead to a change in the questions asked to potential victims to detect it: “Is anyone manipulating or controlling you, even if they would say they are not?” or “Are you in a relationship that is hurting your wellbeing, or in which you feel alone or isolated?” (p. 50).

The selected articles have identified various mechanisms used by perpetrators to exert such psychological coercion on their victims.

The first of these is **gaslighting.** This is present in practically all the selected articles as a mechanism for causing emotional confusion in women and, in many cases, for blaming them for the abuse perpetrated by men, thereby reversing the blame (Arboleda-Trujillo et al., 2025; Daw et al., 2023; Mohd Ali, 2025; Tullio et al., 2021). This gaslighting manipulates the victim’s perception of what they are experiencing, causing them to doubt their memory and their own opinion regarding what is happening (Daw et al., 2023; Isaiah et al., 2024; Lahav, 2021; Zapcic et al., 2024), and leading to so-called brainwashing (Tullio et al., 2021).

One of the mechanisms also highlighted as being used in such coercion is **the alternation of violence and affection** to create emotional dependence (so-called intermittent abuse) (Isaiah et al., 2024). This creates cognitive distortions in the victim that make it difficult for them to perceive and detect the violence, thereby minimising it, whilst also promoting self-blame (Isaiah et al., 2024; Lahav, 2021; Tullio et al., 2021). This mechanism is also employed in serious cases of violation of women’s sexual freedom, as Ahn (2019) describes in her analysis of the coercion suffered by “comfort women” and Japanese soldiers. She also describes the emotional manipulation that created the perception that the perpetrators could be seen as “brutal beasts”, but also as “kind saviours”, due to this alternation between violence and affection.

**Social isolation** is a mechanism that manifests in various ways. One such method involves expressing disapproval during interactions with people whom the abuser deems unsuitable or dislikes (Daw et al., 2023; Mohd Ali, 2025; Tullio et al., 2021). This gradually erodes the victim’s social connections. By severing these support networks, the victim’s emotional dependence on the abuser increases, as does the control exercised over her (Daw et al., 2023). To these mechanisms of coercion, the abuser adds an atmosphere of a false sense of ideal love, in which he is the only necessary relationship, causing the woman to increase her dependence on him even within a short period of time spent with the abuser (Daw et al., 2023).

Another mechanism of coercion is **silence**. This is used as a form of emotional punishment and affective manipulation (Mohd Ali, 2025). A silence that conveys a message to the woman that he is withdrawing his affection from her because of her behaviour, until she changes or he decides it is enough (Daw et al., 2023).

The normalisation of abuse due to the abuser’s **narrative control** over the events that unfold is another highly effective mechanism (Lahav, 2021). This narrative control causes the victim to internalise the abuser’s perspective, and thus normalise and justify it (Lahav, 2023). In the words of Tullio et al. (2021), this involves the perpetrator appropriating the victim’s self-esteem, like a ‘vampire’ who sucks their vital energy. Zapcic et al. (2024) recount experiences of victims who stated that they had no energy or felt unable to stop the abusive situation. This narrative control creates confusion in situations where, when victims attempt dialogue, the abuser shifts the topic to something that blames the woman for the situation. This narrative control also encompasses how **the abuser**, when confronted, **plays the victim** to instil further guilt and submission in the woman, thereby causing her to attribute responsibility for the abusive behaviour she suffers to herself. This creates **induced guilt** (Daw et al., 2023; Lahav, 2021).

Victims are subjected to a **subordination of their autonomy** that strips them of their agency to act and think (Lahav, 2021, 2023). The abuser induces a state of hypervigilance in the victim, causing them to modify their actions as they anticipate the punishment they fear (Lahav, 2023). Furthermore, they are **overburdened with tasks** and domestic control, which leads to the perpetrator taking over the woman’s time, thereby causing her to lose her autonomy and the opportunity to engage in other activities, for herself or with people other than the perpetrator (Mohd Ali, 2025).

The destabilisation of the victim through **lowering self-esteem** or instilling guilt generates fear without the need for physical violence and increases psychological coercion to prevent the victim from leaving (Daw et al., 2023; Isaiah et al., 2024). The victim is belittled and humiliated as a mechanism of coercion, increasing their subjugation (Tullio et al., 2021). Added to this is punishment for any signs the victim may show of independence or independent thought, inducing feelings of shame and guilt (Tullio et al., 2021).

The **idealisation of the abuser**, where the victim believes they cannot live without them, leading to one of the strongest forms of psychological dependence (Isaiah et al., 2024), as a coercive mechanism, is created based on the fear of abandonment and of never finding anyone like them (Tullio et al., 2021).

Bay-Cheng et al. (2018) use the term “intimate terrorism” to define coercive mechanisms that take multiple forms (physical, emotional, and sexual) and generate a form of systematic and continuous subjugation, in which women feel that they are to blame for the situation they are experiencing. One form of coercion is the fear arising from constant persistence, and even the romanticisation of that persistence (Beare & Boonzaier, 2020).

Finally, one of the selected articles, whilst not directly addressing the coercive mechanisms employed by the perpetrator, demonstrates how victims develop traumatic bonds that keep them in the relationship (Effiong et al., 2022). These bonds develop following psychological dynamics of abuse by the perpetrator, which lead the victim to minimise the abuse, experience fear and emotional dependence, and lose autonomy—particularly as a result of prolonged coercion that erodes the woman’s identity: “I cannot make decisions,” “When others ask me how I feel about something, I don’t know”, “I don’t know who I am” (Effiong et al., 2022, p. 3626).

### 3.2. Mechanisms of Coercion in the Victim’s Social Environment

In this case, a total of 11 of the 31 selected articles address the social mechanisms of coercion that perpetuate the manipulation and subjugation of women within the context of abusive relationships.

The analysis identified six key elements relating to this sub-theme that have been examined in the present scoping review. One of these aspects, focusing on the influence of the social context, refers to **economic dependence** (Barnett et al., 2016; Lahav, 2023; McLeod et al., 2019; Arboleda-Trujillo et al., 2025). According to various studies, women’s economic subordination to men influences their subjugation and the perpetuation of gender-based violence. In a sense, it becomes a strategy of control that systematises male domination, which is constructed under the narrative that the person providing financial stability (usually the man) holds the power in the relationship, thereby making it difficult for the other party (usually the woman) to leave. In line with these structural mechanisms, the analysis by Gutzmer et al. (2016) illustrates that the **unequal division of power,** marked by patriarchal norms, favours the perpetuation of the notion that the man is in charge, and that his sexual desires must be satisfied. This cultural framework has a direct impact on the reproduction of dynamics of sexual subjugation to which women who are victims of violence are subjected.

The second element identified in the literature review relates to the **family**. Various studies suggest that the **upbringing** received within the family is a factor explaining the victimisation of women in intimate relationships marked by violence (Chernyak, 2018; Llano-Suárez et al., 2026; Metz et al., 2019; Arboleda-Trujillo et al., 2025). For example, Llano-Suárez et al. (2026) note that the lack of reflection in this context on sex education and equal relationships, as well as the absence of social judgment regarding abusive relationships, may be factors that normalise sexual coercion and its persistence over time. Similarly, Metz et al. (2019) argue that the family environment can become a space in which pressure is exerted to remain in an abusive relationship, even in the face of violence. This type of family pressure reinforces the persistence of such relationships. Ultimately, the family interactions trivialising male dominance represent an intergenerational transmission of domination and may be a factor explaining the subjugation of women and the perpetuation of relationships marked by gender-based violence (Arboleda-Trujillo et al., 2025).

The third element refers to the peer group and friendships. Some studies explore the influence of this aspect on the perpetuation of abusive romantic and sexual relationships. For example, Llano-Suárez et al. (2026) demonstrate that group discourse can have a direct impact on engaging in violent relationships, creating a climate that reinforces and normalises them, whilst also **normalising sexual pressure** within them. Furthermore, friends can also become agents of control and **emotional manipulation**, thereby acting as perpetrators and hindering victims’ ability to leave violent relationships (Daw et al., 2023).

There is a further element—the fourth we have identified—which highlights the stigma that the victim’s social circle constructs around **divorce or separation** (Lipinsky & Goldner, 2026; Mohd Ali, 2025). For example, Mohd Ali (2025), in her qualitative research with women, explains that the community and the context surrounding them can become mechanisms of control, where questioning such a relationship or wanting to leave it can lead to social exclusion and isolation. Similarly, Lipinsky and Goldner (2026), also through a qualitative study with Jewish women in Israel, suggest that the community exerts pressure to continue abusive relationships, so as not to break up the family unit, as taking care of the family is part of the female duties in patriarchal gender roles. Thus, women report a fear of rejection and the stigma that such a separation might provoke, creating, in parallel, a social environment that reinforces their dependence and the impossibility of leaving a violent relationship. Closely linked to this is **the patriarchal interpretation of religious texts**, which is another element that the literature identifies as relevant in sustaining coercive control. In this regard, Mohd Ali (2025) notes that there are certain interpretations of mystical texts, supported by some religious communities, which justify and reinforce male domination. Similarly, a specific interpretation of the hadith, which is a second source of Islamic law and theology after the Quran, is used to demand female submission and downplay men’s ethical obligations.

Finally, the sixth element identified in our analysis highlights that another factor that can foster submission, coercion, and the continuation of an abusive relationship is the creation of a **culture of silence** (Daw et al., 2023; Lipinsky & Goldner, 2026; Schneider, 2018). This culture manifests itself in various ways. For example, Daw and colleagues (2023) highlight the reproduction of **passive solidarity**, where the victim’s social circle remains silent in the face of harassment, making it difficult to break free from the cycle of violence. From another perspective, Lipinsky and Goldner (2026) point out that the establishment of community norms that discredit reporting abuse or leaving a violent relationship perpetuates female submission. Furthermore, this culture of silence sustains coercion and hinders the search for external help. These authors also emphasise that, even when the community is aware of the coercion, they do not intervene, which leads to the victim’s isolation and the normalisation of violence.

### 3.3. The Influence of Gender Socialisation on Legitimising the Interpersonal and Social Power Dynamics That Subjugate Women

To respond to this section, 17 articles out of the 31 have been taken into account. While most articles somehow imply or depart from the idea that gender socialisation is influencing the subjugation of women, only these articles directly address socialisation and/or sexual scripts as the research object or main focus. The articles emphasise the important role of a gendered socialisation that takes place in the **primary socialisation,** through socialising institutions such as the family (Barnett et al., 2016; Bhandari, 2024; Lipinsky & Goldner, 2026; Mohd Ali, 2025), and religion (Lipinsky & Goldner, 2026; Mohd Ali, 2025), as well as the **cultural aspects** that mark socialisation (Bard Wigdor, 2016; Mitchell & Bennett, 2020; Šudila Žilinská & Bianchi, 2025). Victims who ask their natal families for help in addressing violent marriages are often turned down, and their experiences are being normalised (Bhandari, 2024; Lipinsky & Goldner, 2026; Mohd Ali, 2025). They are told by other men and women that it is a woman’s task to take care of the family, regardless of the behavior of the husband, even if he is violent. Female subjugation is thus transmitted from one generation to the next through reproducing a **patriarchal gender socialisation**.

Research on religious socialisation suggests that **some forms of community-based religious socialisation** may reinforce patriarchal gender roles linked to male authority and female subordination. However, the literature also indicates that these norms are rooted in wider patriarchal social structures rather than in religion per se. This is reflected in studies on Christian Domestic Discipline (Deshotels et al., 2019), the Jewish Ultra-Orthodox community (Lipinsky & Goldner, 2026), and Malay-Muslim communities (Mohd Ali, 2025). In fact, all three studies mention that it is the misinterpretation by specific communities of religious texts to subjugate women. While the Christian Domestic Discipline community refers to individuals following a certain lifestyle, sharing their experiences in online forums (Deshotels et al., 2019), the Jewish Ultra-Orthodox community analysed by Lipinsky and Goldner (2026) comprises approximately 12% of the Israeli population that lives mainly isolated from the larger Israeli society. Both studies indicate a presence of high control mechanisms. In all three cases, women’s agency is limited, and subordination, including physical and psychological abuse, may be legitimised through specific religious interpretations and community norms. These dynamics, however, are not exclusive to religious groups, but also emerge across other cultural and socialisation contexts. Different studies reinforce this understanding of **cultural norms** that distinguish gendered roles and their impact on **reproducing male dominance and female subjugation** (Arboleda-Trujillo et al., 2025; Barnett et al., 2016; Bard Wigdor, 2016; Mitchell & Bennett, 2020; Šudila Žilinská & Bianchi, 2025).

This form of control of women from diverse social spheres becomes evident in diverse studies. In this sense, Mitchell and Bennett (2020) show that women, rather than looking at their own needs and desires, have to balance their **sexual behaviour** according to either adhering to social norms of no premarital sex or fulfilling the expectations of romantic partners to engage in sex. These studies align with stereotypical, patriarchal gender roles, where women are supposed to cater to men’s needs, including men’s sexual needs, while disregarding any personal needs or desires.

The reviewed articles also point to the **secondary socialisation agents**, such as the **peer group** and **media** (Hill et al., 2023; Llano-Suárez et al., 2026; Rodgers et al., 2023). One way of inscribing patriarchal gender roles in secondary socialisation, especially in terms of sexual roles, takes the form of **sexual scripts,** giving practical examples of sexual encounters between men and women. These scripts often portray a highly patriarchal and gendered understanding of sexual relationships. Two of the studies reviewed focused on the role of **music** in perpetuating the subjugation of women. Analysing the lyrics of popular rock and metal music, Hill et al. (2023) evidence the normalisation of sexual violence by representing mechanisms such as gaslighting, objectification, infantilisation, or the simple absence of female perspectives on relationships or sexual encounters, among others. Rodgers et al. (2023) analyse the impact of these scripts portrayed in music videos on the likelihood of seeking consent, adhering to sexual consent decisions, and refusing unwanted sexual encounters. They found that music videos that portray a subjugation of women reinforce the belief in stereotypical sexual scripts and can impact sexual consent, both for men perpetrating violence who do not seek consent, as well as for women who can be at risk of not refusing unwanted sexual encounters.

**Pornography** is another form of sexual script that is analysed in the studies reviewed here and is particularly important as it portrays explicit forms of sexually subjugating women. The studies point to the idea that pornography has become a source of sexual education for younger generations, influencing especially young men’s expectations of sexual encounters, as well as their desires and preferences (Llano-Suárez et al., 2026; Vera Cruz & Sheridan, 2022). According to Vera Cruz and Sheridan (2022), young men are more likely to be drawn to consume pornography and experience arousal from it, while women are less likely to consume it on a voluntary and sexual basis. Yet, both men and women report having violent sexual encounters inspired by pornography. Thus, while 77.6% of male study participants responded to occasionally (one in five sexual relationships) inflict violence on their sexual partners, and 45.8% enjoying dominating their partners, female participants reported the highest numbers of negative consequences, such as lack of self-confidence and low self-esteem (63.1%), as a result of violent sexual encounters inspired by pornography (Vera Cruz & Sheridan, 2022). Although these results do not demonstrate a correlation or causal link between submissive behaviour in pornography and violence against women, they provide valuable information to bear in mind in our analysis.

Another aspect influenced by gendered socialisation that emerges from the research reviewed is **consent** (Beare & Boonzaier, 2020; Fanghanel, 2025; Gomez-Pulido et al., 2024). The authors emphasise that the **lack of a clear definition of consent** complicates the understanding of sexual relationships in myriad ways. On a **personal level,** women can feel fear, shame and ridicule for consenting or not to certain sexual encounters that align with the different social expectations towards women (Gomez-Pulido et al., 2024). Women are seen as gatekeepers of sexual advances, whether they consent or not. Yet, consent is deeply marked by patriarchal norms, which are reflected in women’s diverse definitions of consent. Beare and Boonzaier (2020) identify three types of consent: (1) consent as a woman’s call; (2) consent without desire; and (3) consent with willingness. The first two respond to patriarchal perceptions of women’s duty to cater to men’s needs. It shows how social expectations of gender roles lead women to engage in unwanted sexual encounters just because that is what they feel is expected from them (Beare & Boonzaier, 2020; Duby et al., 2023; Gomez-Pulido et al., 2024). Some hope they might end up enjoying it, although, in the first place, they do not want sex (Gomez-Pulido et al., 2024), evidencing the internalised patriarchal sexual scripts of women rejecting sexual advances to end up consenting and enjoying them. The third type of consent, though, refers to a mutual consensus of the participants in the sexual encounter. Thus, breaking with the patriarchal idea of women as gatekeepers, since consent needs to be mutually constructed. This is also a conclusion that Fanghanel (2025) reaches in her analysis of how consent is used in court cases of violent sexual games gone wrong. She points out that the patriarchal interpretation of the sexual interactions tends to favour the perpetrator by implying consent of the victims to rough sex and the resulting injury or death. As a result, perpetrators are being given lower sentences or even absolved. This indicates the implications of patriarchal gender norms and socialisation going far beyond the individual level, but also affecting the **legal level**. Fanghanel (2025) claims that the lack of clear definitions of how consent is constructed allows for an interpretation under patriarchal social norms.

Both the gendered socialisation and the sexual scripts present in our everyday lives are replete with **social dynamics that subjugate women**. Oftentimes, the presence of these dynamics and sexual scripts is rendered normal, rather than problematic (Bhandari, 2024; Duby et al., 2023; Hill et al., 2023; Šudila Žilinská & Bianchi, 2025). At the same time, socialisation in gender roles and sexual scripts leads to an **internalisation of the subjugating dynamics**, making it difficult for women to identify their own needs and desires in a sexual encounter, as well as identifying and tackling subjugating dynamics appropriately (Beare & Boonzaier, 2020; Bhandari, 2024; Mohd Ali, 2025; Šudila Žilinská & Bianchi, 2025). Thus, engaging in sexual encounters according to the prescribed gender roles simultaneously reproduces them, reinitiating the cycle of gender socialisation in **power dynamics** that subjugate women.

## 4. Discussion

When trying to address violence against women from different social spheres, such as the social services, police investigations, legal systems, medical care, or even research on the topic, often the question is posed: “Why do women stay with their abuser?” The idea that women can freely choose to stay in, or leave, an abusive relationship has been a departure point for research and action on addressing violence against women. Thus, economic aspects were taken into account, emphasising the fact that women might not always have the economic means to leave an abusive partner. Support systems have been established to counter economic difficulties, provide housing, counter social isolation of women, etc. Regardless of the crucial contribution of these support systems, they do not address the patriarchal system in which violence against women takes place. As MacKinnon (1989) argues, patriarchy functions as a system of power that structures social, legal and political relations in ways that normalise and sustain male dominance, including violence against women. From this feminist perspective, violence is not an isolated or individual phenomenon, but a systemic expression of gender inequality embedded in social institutions. Already in the 1980s, Gerda Lerner emphasised that we need to put women at the center to readjust society to be inclusive of all genders. So, when analysing violence against women, the focus needs to be put on women, but not to place responsibility for *remaining* in a violent relationship on them. The question should rather be: “What is it that *keeps women trapped* in an abusive relationship?” Placing responsibility on the abuser, what coercion mechanisms does the abuser use to dominate and keep the women he abuses in a state of subjugation?

Our scoping review contributes to an overview of common coercion mechanisms of female subjugation present across cultures and societies from different places in the world, and in diverse social settings, including social, religious, and cultural minorities in their respective countries. The settings reviewed include sexual encounters, romantic relationships, and marriages, as well as partner relationships in coercive groups, such as specific religious groups. The literature reviewed helps provide a systematic overview of a broad set of mechanisms that are employed by perpetrators, by the social environment of victims, and as well in the larger society. The studies reveal that perpetrators deliberately employ and combine diverse strategies to manipulate women into obedience. These include both physical and psychological violence, such as gaslighting, intermittent abuse, social isolation, silence, interpretative or narrative control over the events to normalise abuse, and invert responsibility to blame the victim for the situation. Also, the subordination of a woman’s autonomy is used by perpetrators to control not only their actions, but also their thinking. This is sometimes paired with an overburdening of tasks that leaves victims no space or time for independent thought or action (Mohd Ali, 2025). Last but not least, a common strategy employed by perpetrators is the destabilisation of victims through humiliation, degradation, etc., to purposefully erode self-esteem in the victim. The perpetrator portrays himself as the “savior” for whom he considers an “unworthy” human being. All these mechanisms have a lasting impact on the victims, meaning that they can persist beyond coercive situations. Victims can be more or less affected by these subjugating and coercive mechanisms, and they can have more or less agency to actively free themselves from an abusive situation. But it is necessary to acknowledge that, under these circumstances, decisions cannot be taken freely. These mechanisms undermine the conditions for free choice and can even undermine a victim’s agency beyond the abusive situation.

Moreover, the mechanisms employed by a perpetrator are reinforced by social mechanisms rooted in a patriarchal society that perpetuate male dominance. The scoping review identified several social mechanisms: economic dependence and an unequal division of power, patriarchal family traditions and norms, patriarchal interpretations of religious texts, as well as pressure from the peer group and friends. As such, the interactions that women have with their friends, peers, and family members influence their perception of the abuse suffered. In this sense, another important social mechanism fostering female subjugation is the culture of silence, referring to the fact that situations of violence against women are often overlooked, not taken seriously, or played down. If the bystanders of particular violent situations remain silent, the subjugation is being normalised, as it does not appear to be problematic. Rather, the fact of verbally bringing doubts about the abuse up by a victim with her family members or peers can be considered problematic. If family or peers reject complaints or blame the victim for not appropriately fulfilling her role as a woman in the patriarchal society, they are reinforcing the social norms of subjugating women. These everyday practices and interactions with the close environment of victims are crucial in stabilising subjugation mechanisms employed by the perpetrator, as they justify it and allow for it to happen. We thus call them social coercion mechanisms.

Altogether, the psychological coercion mechanisms employed by the perpetrator and the social mechanisms that sustain women’s subjugation contribute to the enactment and reproduction of gendered power norms. A third crucial mechanism is therefore gendered socialisation, through which unequal expectations about masculinity, femininity, sexuality, obedience, and authority are learned and normalised. The studies reviewed showed that, across geographically, culturally, and religiously diverse populations, these norms are transmitted through both primary and secondary socialising agents such as the family, peer groups and friends, religious communities, media and music, among others. These socialising processes may encourage women to internalise expectations of obedience, passivity, caretaking, neglecting their own desires and needs, while simultaneously normalising men’s entitlement to control, service, sexual access, and authority. Moreover, sexual scripts embedded in romance, intimacy, cultural traditions, religious expectations, and pornography may obscure coercion by framing it as consent, desire, intimacy, or ordinary relationship dynamics. In this sense, gendered socialisation does not merely reflect patriarchal norms but helps reproduce the conditions under which coercion can be normalised and rendered less visible.

Taken together, these three spheres—the perpetrator’s coercive mechanisms, the social environment’s reinforcement of abuse, and gendered socialization—interact with and reinforce one another. They contribute to normalising unequal power dynamics and may restrict women’s agency, autonomy, and capacity for resistance. It shapes them into compliant, self-sacrificing actors responsible for caregiving duties. Staying in an abusive relationship is therefore closely tied to the interaction between these spheres, rather than to an individual victim’s free or isolated choice. Coercive control by an individual perpetrator is more likely to be effective when the surrounding social environment remains silent, minimises abuse, blames the victim, admires or protects the perpetrator, or fails to challenge unequal power relations. At the same time, broader socialisation processes may make these dynamics appear natural, acceptable, or difficult to identify as abuse. Although important progress has been made in challenging unequal gender norms and diversifying relationship models across societies, the findings of this review suggest that psychological and social mechanisms of coercion must be taken seriously when analysing victims’ possibilities for action, resistance, and exit.

Although the phenomena explored in this review include different typologies of violence against women and forms of discrimination that lead to domination, such as intimate partner violence, coercive control, traumatic bonding, sexual coercion, pornography, sexual scripts, and culturally grounded gender hierarchies, they share common social and structural elements. These elements are analytically connected through their role in producing, normalising, and maintaining gendered power asymmetries and coercive dynamics against women. While these forms of violence differ in their specific contexts, mechanisms of control, and profiles of victims and perpetrators, this review highlights how individual coercion is embedded in wider social processes. The scoping review shows how socialisation, everyday interactions, institutional arrangements, cultural narratives, religious interpretations, and the immediate social environment can reinforce coercive dynamics and make it more difficult for women to break free from abusive situations. Therefore, rather than asking why women stay, the analysis should focus on the psychological, relational, social, and structural mechanisms that constrain their freedom to leave.

### 4.1. Practical Implications

This scoping review also has several practical implications for professionals, policymakers, and educators addressing violence against women, as well as for society as a whole. Shifting the focus from women’s decision-making to the coercive mechanisms employed by perpetrators implies that practitioners in social services, law enforcement, and the legal system should be trained to identify patterns of coercive control and psychological manipulation, rather than relying on assumptions of ‘free choice’.

This perspective is also essential in educational contexts. Violence prevention programmes must avoid reproducing victim-blaming narratives, and instead place responsibility on the perpetrator, while also addressing the conscious and unconscious social complicities that sustain abuse. A feminist analysis of patriarchy should underpin educational interventions, recognising that violence against women is embedded in broader systems of gender inequality.

In this sense, acknowledging the role of gendered socialisation and patriarchal norms highlights the importance of preventive interventions, particularly in education and media, aimed at transforming gender norms, deconstructing harmful sexual scripts, and promoting egalitarian relationship models. On this basis, it becomes possible to incorporate a critical understanding of psychological and social coercion mechanisms into prevention and intervention strategies.

Finally, awareness of social coercion mechanisms highlights the need to engage not only victims, but also their social environments—including family members, peers, and wider communities—to challenge and transform norms that normalise or silence violence against women.

### 4.2. Limitations

While the studies focus on populations from all continents, analysing heterosexual relationships among adults that portray male dominance and female subjugation, the setting that these studies analyse mainly refers to romantic, sexual relationships and coercive family systems, such as marriages, romantic relationships, and sexual relationships. We were initially expecting results of diverse settings of abusive relationships, as they can also occur in coercive group organisations, beyond family systems. The articles identified only shed light on the partnership setting and therefore indicate a potential gap in the literature about mechanisms of female subjugation in coercive groups outside of family systems. Moreover, the studies each focus on a specific subset of the population. Thus, generalisations to the greater population cannot be inferred. However, the scoping review extracted common traits that appeared among all these studies.

Another limitation of the present review is the focus on structural aspects of patriarchal societies, rather than on agency and how individuals manage to resist or escape coercive situations or groups. While a focus on women’s agency and potentials for resistance are crucial to strengthen women’s ability to exit those environments, potentially, our focus aims at re-centering the attention away from women as actors to be responsible for their experience, that sometimes ends up blaming victims, to addressing the problem of violence against women from a feminist perspective on abusive relationships that takes the patriarchal context and structures of violence against women into account.

The studies reviewed include minority groups, but do not engage in the intersectionality of victims’ experiences. Future research should address this limitation by engaging with an intersectional perspective, as proposed by Crenshaw (1989), to better account for how overlapping systems of power and inequality shape diverse experiences of subjugation across different groups of women.

This scoping review focuses on the mechanisms that keep women trapped in abusive relationships. It does not shed light on how these dynamics can be changed. So, further research could depart from the possibilities of education, internal reflection, and taking action in our own lives as well as for the larger societies we participate in (Hooks, 2000b) to investigate what are the mechanisms or strategies that encourage change and overcoming female subjugation.

Lastly, we reviewed psychological and social coercion mechanisms that entrap victims, with a focus on how these mechanisms are used by perpetrators and reinforced in patriarchal social settings. This focus disregards the long-lasting impact that psychological and social coercion mechanisms can have on victims. Also considered are the survival strategies of victims in abusive situations; these strategies can persist over time and even beyond coercive settings, creating an internal entrapment of victims. This would be another dimension, especially of the psychological mechanisms, to consider in future research on the topic.

## 5. Conclusions

The present study shows that there are patterns across cultures and regions that indicate that it is not the victim who ‘decides’ to stay in an abusive relationship because of, for example, economic dependence, attachment, or attraction to a violent man. Rather, the perpetrator exercises coercive actions against the victim from a position of power, shaped by patriarchal social dynamics. This power may also be reinforced by the complicity, admiration, silence or lack of questioning of those around him, such as family members, friends, colleagues or other members of the immediate social environment. In this sense, male perpetrators may employ mechanisms of manipulation and control that are not only individual but also socially reinforced through everyday processes of primary and secondary socialisation across cultures and social backgrounds.

Hence, when attempting to address violence against women, the focus needs to shift to acknowledging these mechanisms, placing the perpetrator at the centre of responsibility for women’s subjugation, rather than attributing blame to the victims, and creating the conditions for women to free themselves from that subjugation. The problem does not lie in Bluebeard’s wives—those who are seemingly able to leave do not—but in the mechanisms of manipulation and coercion exercised over them. It is in overcoming these conditions that the possibility of their freedom resides.

## Figures and Tables

**Table 1:** Keywords used for the identification of relevant studies.

(“women” OR “girl” OR “female”) AND (“gender based violence” OR “gender violence” OR “intimate partner violence” OR “sexual violence”)	AND (“coercion” OR “control”)
	AND “psychological manipulation”
	AND “traumatic bonding”
	AND “gender socialisation” OR “gender scripts”

**Table 2:** Inclusion and exclusion criteria.

**Inclusion Criteria**
Peer-reviewed empirical, theoretical, or conceptual articles. The article examines psychological and/or social coercion/control mechanisms related to staying in or difficulty leaving a violent relationship/context (e.g., coercive control, isolation, humiliation, threats/intimidation, manipulation/gaslighting, intermittent reinforcement, imposed dependency, learned helplessness, erosion of autonomy/identity, gender socialisation, interpersonal power). The article links these mechanisms to outcomes such as remaining/staying, returning, barriers to leaving/escaping, submission, or reduced agency/autonomy (or equivalent constructs). Include articles that study the adult female population. Eligible contexts include intimate and sexual relationships, family systems, high-control groups, and organisational/institutional settings, where sustained coercion/control is present. Articles about how gender socialisation or gender scripts influence and legitimise the interpersonal and social power dynamics that subjugate women.
**Exclusion Criteria**
Studies that address violence/abuse, but do not analyse coercion/control mechanisms (e.g., prevalence-only, descriptive profiles, or broad correlates without coercive processes). Studies that do not link coercion/control to staying, returning, difficulty leaving/escaping, submission, reduced agency/autonomy, or equivalent outcomes. Studies offering material/external-only explanations (economic dependence, lack of resources, institutional barriers, lack of support) without psychological/social coercion mechanisms. Studies focused on isolated incidents or violence without a pattern of sustained control/power dynamics. Studies are not about violence from men against women. Exclude articles that focus on girls/minors only.

**Table 3:** Summary of results.

Authors	Title	Year	Methodology and Participants	Psychological Coercion Mechanisms from an Individual Abuser	Social-Environmental Coercion Mechanisms	Gender Socialisation and Sexual Scripts
Ahn, Yonson (Ahn, 2019)	Yearning for affection: Traumatic bonding between Korean ‘comfort women’ and Japanese soldiers during World War II	2019	South Korea and Japan. (Tokyo, Seoul, Incheon, and Ulsan). Qualitative study based on a combination of in-depth interviews between 1992 and 1996, and published/second-hand interview sources. 9 participants. 4 Japanese veterans and 5 Korean “comfort women” victim-survivors.	It describes psychological coercion tactics such as gaslighting, humiliation, emotional manipulation, and intimidation, amongst others.	No relevant information	No relevant information
Arboleda-Trujillo, M.A.; Lennon, S.E.; Pacichana-Quinayaz, S.G.; Fandiño, C.A.; Gutiérrez, M.I. (Arboleda-Trujillo et al., 2025)	Intimate Partner Violence: The Perspective of Men Living in Two Municipalities in Valle del Cauca, Colombia	2025	Colombia. Open-questions interviews. An exploratory qualitative study using focus group discussions. The study used a socioecological approach; six focus groups were conducted. Men aged over 18, residents of Comuna, Cali, or Tuluá. They were recruited through community leaders and snowball sampling; the sample also included local community leaders.	It describes male discourses that normalise psychological coercion.	This article describes economic domination as a structural element, functioning as a mechanism for coercion and the maintenance of submission.	Describe the reproduction of patriarchal gendered norms.
Bard Wigdor, Gabriela (Bard Wigdor, 2016)	Aferrarse o soltar privilegios de género: sobre masculinidades hegemónicas y disidentes	2016	Argentina, specifically Córdoba city (Capital de Córdoba). Theoretical and empirical approach. The article draws on international masculinity studies and on qualitative interviews conducted in 2015. 15 interviews with men: 5 in-depth interviews and 10 semi-structured interviews. Men aged 20 to 40 from the popular sectors and the professional sectors. The broader material also included activists from anti-patriarchal men’s organisations, groups on “new fatherhoods,” and professional men in Córdoba who did not participate in such organisations.	No relevant information	No relevant information	Describes patriarchal norms and especially masculinities.
Barnett, J.P.; Maticka-Tyndale, E.; Kenya, T. (Barnett et al., 2016)	Stigma as Social Control: Gender-Based Violence Stigma, Life Chances, and Moral Order in Kenya	2016	Kenya, specifically Nakuru, Nairobi, and Meru. Qualitative study using focus groups and one-on-one interviews. Data were collected through focus group discussions with women survivors and interviews with close others and key informants. 59 participants in total: 40 women survivors in focus groups, 11 close others, and 8 key informants. Primary participants: women survivors of intimate partner violence/spousal GBV Close others: family members or close friends referred by the women Key informants: people working with or knowledgeable about spousal GBV, including paralegals, lawyers, chiefs, a counselor, and a female police officer.	No relevant information	This article describes economic domination as a structural element, functioning as a mechanism for coercion and the maintenance of submission.	Describes patriarchal marital norms.
Bay-Cheng, Laina Y.; Maguin, Eugene; Bruns, Anne E. (Bay-Cheng et al., 2018)	Who Wears the Pants: The Implications of Gender and Power for Youth Heterosexual Relationships	2018	United States. Participants were U.S. residents recruited through Amazon Mechanical Turk (MTurk). Convergent mixed-methods study using a web-based Digital Sexual Life History Calendar (d/SLHC). The study combined quantitative ratings of relationships with qualitative open-ended accounts, and the quantitative data were analysed with linear mixed modeling, while the qualitative data were examined through directed content analysis. 114 participants were included in this analysis. They provided data on 395 heterosexual relationships. Emerging adults aged 18–25, all cisgender, including 59 women and 55 men.	It describes mechanisms of psychological coercion as a means of controlling one’s partner.	No relevant information	No relevant information
Beare, Kayla; Boonzaier, Floretta (Beare & Boonzaier, 2020)	South African women’s constructions of sexual consent	2020	South Africa, at a university in Cape Town. Qualitative study using a feminist framework and focus group discussions. Data were analysed with Foucauldian Discourse Analysis (FDA). 25 participants. University students with experience of romantic heterosexual relationships. Most participants identified as cisgender women (24), and one participant identified as genderqueer. Ages ranged from 19 to 38 years.	It describes mechanisms of psychological coercion, such as pressure or fear.	No relevant information	Describes how gender roles impact consent.
Bhandari, Shreya (Bhandari, 2024)	Exploring Intervention with South Asian Women in the United States Experiencing Domestic Violence	2024	United States. Qualitative study based on in-depth telephone interviews. The study used a convenience sample, and the interviews were analysed using thematic analysis within the CDC ecological model framework. 20 participants. South Asian women in the U.S. who had experienced domestic violence. Participants were aged 27 to 68 and were recruited through one South Asian women’s organisation and snowball sampling. They came from different South Asian backgrounds, including India, Pakistan, Nepal, Bhutan, Bangladesh, the UK/India, and the U.S.	No relevant information	No relevant information	Describes gender roles in marital relationships and family socialisation.
Chernyak, E. (Chernyak, 2018)	Intimate partner violence in Tajikistan: Risk and protective factors	2018	Tajikistan. Quantitative secondary data analysis based on the 2012 Tajik Demographic and Health Survey (TjDHS). The study used SPSS 21.0 and STATA 13, including univariate descriptive analysis and multilevel regression models for survey data, with binomial and ordered logistic regression. 4401 women. Ever-married or cohabiting women aged 15–49 who completed the domestic violence module of the TjDHS.	No relevant information	This article examines the pressure from family to stay in a toxic relationship and how this affects one’s ability to leave it.	No relevant information
Daw, Jennifer; Halliwell, Gemma; Hay, Susie; Jacob, Suzanne (Daw et al., 2023)	You don’t notice it, it’s like boiling water: Identifying psychological abuse within intimate partner relationships and how it develops across a domestic homicide timeline	2023	United Kingdom. The study focuses on survivors in the UK and on intimate partner relationships in the context of domestic homicide risk. Qualitative survivor-led study using secondary analysis of semi-structured interview transcripts. The authors used deductive Framework Analysis, guided by the Domestic Homicide Timeline (DHT). 12 participants. Women survivors of intimate partner abuse/nonviolent coercive control, all aged over 18 at interview; most were White British.	It describes non-violent psychological coercion tactics such as manipulation, isolation or the confusion of victims.	This research identifies how the victim’s social circle fosters a culture of silence that makes it difficult for her to leave the abusive relationship.	No relevant information
Deshotels, Tina Hebert; Forsyth, Craig J.; Earwood, Stephanie; New, BreeAnna; Fulmer, Jennifer (Deshotels et al., 2019)	For HE tells me so: Techniques of neutralization applied to Christian domestic discipline	2019	United States. The authors were affiliated with universities in Alabama, Louisiana, and New Mexico, and the study analysed online material from Christian Domestic Discipline communities. Qualitative document/online content analysis. The study analysed publicly available anonymous internet sources, specifically 16 Christian Domestic Discipline websites and 35 testimonials, using a coding scheme based on techniques of neutralisation. 51 data sources in total: 16 websites and 35 testimonials. The testimonials were written by self-identified Christian Domestic Discipline practitioners, specifically male “Heads of Household” and female partners/wives.	No relevant information	No relevant information	Analyses Christian Domestic Discipline.
Duby, Zoe; Bergh, Kate; Jonas, Kim; Reddy, Tarylee; Bunce, Brittany; Fowler, Chantal; Mathews, Catherine (Duby et al., 2023)	Men Rule horizontal ellipsis this is the Normal Thing. We Normalise it and it’s Wrong: Gendered Power in Decision-Making Around Sex and Condom Use in Heterosexual Relationships Amongst Adolescents and Young People in South Africa	2023	South Africa, in six districts across six provinces: Western Cape, KwaZulu-Natal, Mpumalanga, North West, Eastern Cape, and Free State. Mixed-methods study combining a cross-sectional telephone survey with qualitative in-depth interviews. Survey data were analysed using multinomial regression; qualitative data were analysed through iterative thematic analysis. Survey: 515 AGYW. Qualitative interviews: 50 AGYW and 9 male partner/peer respondents. Adolescent girls and young women (AGYW) aged 15–24, plus 9 male partner/peer respondents aged 18+.	No relevant information	No relevant information	Describes influence of patriarchal norms on the decision-making of women about sexuality.
Effiong, James Edem; Ibeagha, Peace N.; Iorfa, Steven Kator (Effiong et al., 2022)	Traumatic bonding in victims of intimate partner violence is intensified via empathy	2022	Nigeria, specifically Awka, Anambra State, and Lagos. Quantitative study using purposive sampling and self-report questionnaires. Data were analysed using the Hayes regression-based PROCESS macro to test mediation. 345 participants. Women victims of intimate partner violence recruited from two domestic/sexual violence response centres in Nigeria; ages 18–61.	It describes how victims develop traumatic bonds that perpetuate the coercive relationship.	No relevant information	No relevant information
Fanghanel, Alexandra (Fanghanel, 2025)	Queerying consent: Romantic relationship scripts, rape myths, and the ‘sex game gone wrong’	2025	England and Wales/UK. The cases analysed were limited to English and Welsh jurisdiction. Qualitative legal case analysis based on in-depth analysis of Crown Court case transcripts. The author identified cases through LexisNexis, WestLaw, Law Pages, media searches, and triangulation with “We Can’t Consent to This”, then analysed the judge’s summing-up statements. 14 legal cases. Court cases involving women killed or injured by men where a claim was made that the relevant sexual acts were consensual.	No relevant information	No relevant information	Analyses the impact of patriarchal norms on consent defined in legal proceedings.
Gomez-Pulido, E.; Garrido-Macías, M.; Miss-Ascencio, C.; Expósito, F. (Gomez-Pulido et al., 2024)	Under the Shadows of Gender Violence: An Exploration of Sexual Consent through Spanish University Women’s Experiences; Bajo la sombra de la violencia de género: una exploración del consentimiento sexual a través de experiencias de mujeres estudiantes universitarias españolas	2024	Spain, specifically the University of Granada. Mixed-methods research based on two independent studies. Study 1: 308 Spanish female university students. Quantitative cross-sectional survey. Aged 18–44, identified as heterosexual or bisexual, and had at least one sexual relationship with a man. Study 2: 8 Spanish female university students. Qualitative study exploring personal narratives about consent.	No relevant information	No relevant information	Describes the interplay among sexual scripts and consent.
Gutzmer, K.; Ludwig-Barron, N.T.; Wyatt, G.E.; Hamilton, A.B.; Stockman, J.K. (Gutzmer et al., 2016)	“Come on Baby. You Know I Love You”: African American Women’s Experiences of Communication with Male Partners and Disclosure in the Context of Unwanted Sex	2016	United States, specifically San Diego, California. Qualitative study using semi-structured in-depth interviews. Data were analysed using a systematic inductive analytic approach with a hierarchical coding scheme. 19 participants. Sexually active African American women, aged 18–44, living in San Diego County, who reported at least one incident of sexual coercion by a current or former intimate male partner since age 18.	No relevant information	This article describes power relationships as a structural element, functioning as a mechanism for coercion and the maintenance of submission.	No relevant information
Hill, Rosemary Lucy; Richards, Daisy; Savigny, Heather (Hill et al., 2023)	Normalising sexualised violence in popular culture: eroding, erasing and controlling women in rock music	2023	United Kingdom, based on the U.K. Official Rock and Metal Chart. Mixed qualitative and quantitative content analysis of popular music. The authors sampled chart-topping rock and metal songs at 5-year intervals from 1995 to 2015, coded lyrics, artwork, and music videos using a coding frame on sexual violence, and then conducted discourse analysis. No human participants. 60 songs. The units of analysis were No. 1 rock and metal singles from the U.K. Official Chart, representing mainstream rock/metal songs across several subgenres.	No relevant information	No relevant information	Analyses the presence of sexual scripts subjugating women in music.
Isaiah, Uwemedimo S.; Effiong, James E.; Udokang, Innih; Ogwuche, Samson; Udoukok, Emekubong N.; Iorfa, Steven Kator (Isaiah et al., 2024)	Need for closure is linked with traumatic bonding among victims of intimate partner violence (A mediatory approach)	2024	Nigeria, specifically, participants were recruited from Awka, Anambra State, and Lagos. Quantitative study using purposive sampling. Data were collected with self-report questionnaires, and the analysis used the Hayes regression-based PROCESS macro to test mediation. 345 participants. Women who were victims of intimate partner violence, recruited from two domestic/sexual violence response centers in Nigeria. Their ages ranged from 18 to 61 years.	It describes mechanisms of psychological coercion such as intermittent reinforcement, cognitive distortions, and the undermining of self-esteem, amongst others.	No relevant information	No relevant information
Lahav, Yael (Lahav, 2023)	Hyper-Sensitivity to the Perpetrator and the Likelihood of Returning to Abusive Relationships	2023	Israel. Quantitative cross-sectional study using an online survey. Participants were recruited through a Facebook advertisement, and the study used a convenience sample. 258 participants. Adult Israeli women in Israel who had left their abusive intimate partners and reported a history of intimate partner violence.	Describes the mechanism of identifying with the aggressor as emotional coercion.	This research identifies how the victim’s social circle fosters a culture of silence that makes it difficult for her to leave the abusive relationship.	No relevant information
Lahav, Yael (Lahav, 2021)	Painful bonds: Identification with the aggressor and distress among IPV survivors	2021	Israel. Quantitative cross-sectional study using an online survey conducted through Facebook recruitment and administered via Qualtrics. The study used a convenience sample. 297 participants. Adult Israeli Jewish women living in Israel aged 18–78 who reported current or previous intimate partner violence. 68 reported current IPV, and 229 reported past IPV.	Describes the mechanism of identification with the aggressor resulting from prolonged coercive dynamics.	No relevant information	No relevant information
Lipinsky, Aiala Szyfer; Goldner, Limor (Lipinsky & Goldner, 2026)	Antecedents, Characteristics, and Dynamics of IPV in the Israeli Jewish Ultra-Orthodox Community: A Cultural Exploration	2026	Israel (JUO) community. Qualitative study using semi-structured interviews based on the Clinical Ethnographic Narrative Interview (CENI) approach. The interviews were analysed using an Interpretive Phenomenological Analysis (IPA) approach. 15 participants. Israeli Jewish Ultra-Orthodox women (JUO) community. Aged approximately 30 to 55, who were currently living with or separated/divorced from abusive partners and had experienced intimate partner violence.	No relevant information	This article examines two factors, linked to social pressure, that make it difficult to leave an abusive relationship: the stigma surrounding divorce and separation, and the culture of silence.	Describes religious and family socialisation in patriarchal gender norms
Llano-Suárez, Andrea; Lana, Alberto; Fernandez-Feito, Ana (Llano-Suárez et al., 2026)	Intimate partner sexual violence in young people: a qualitative study	2026	Spain, specifically a public university in northern Spain (University of Oviedo). Qualitative descriptive phenomenological study. Data were collected through 17 semi-structured individual interviews and 4 focus groups, and analysed through content analysis using MAXQDA 2020. 37 participants, 17 interviews, and 4 focus groups. Male and female undergraduate students, aged 18–26, from 15 degree programs, who had been in a relationship of at least one month and had engaged in sexual intercourse.	No relevant information	This article suggests that peer groups and friendships can influence a person’s decision to remain in a toxic relationship. The normalisation and legitimisation of sexual pressure within these interactions is a factor that can lead to the subjugation being taken for granted.	Analyses patriarchal norms and socialisation in it.
McLeod, David Axlyn; Sharp, Susan F.; Gatlin, Leah; Jones, Melissa S. (McLeod et al., 2019)	No Idle Threat: Coercive Control and Enacted Violence in the Pre-Prison Relationships of Incarcerated Women	2019	United States, specifically Oklahoma. Quantitative study using a stratified random sample of incarcerated women in three women’s correctional facilities. Data were collected through a self-administered questionnaire using items from the Coercive Control Measure and the Revised Conflict Tactics Scales (CTS2). 367 participants. Incarcerated adult women in Oklahoma prisons, reporting on their intimate relationships in the 12 months prior to incarceration.	No relevant information	This article describes economic domination as a structural element, functioning as a mechanism for coercion and the maintenance of submission.	No relevant information
Metz, Claire; Calmet, Jeremy; Thevenot, Anne (Metz et al., 2019)	Women Subjected to Domestic Violence: The Impossibility of Separation	2019	France. Qualitative study based on semi-directive interviews. The data were analysed through both quantitative text analysis using ALCESTE software and qualitative content/discourse analysis. 30 participants. Women victims of domestic violence who had separated from their violent partner. The sample included women in shelters/reintegration centers, women from the general population, and women connected through an association supporting women from Turkey.	No relevant information	This article examines the pressure from family to stay in a toxic relationship and how this affects one’s ability to leave it.	No relevant information
Mitchell, Elke; Bennett, Linda Rae (Mitchell & Bennett, 2020)	Pressure and Persuasion: Young Fijian Women’s Experiences of Sexual and Reproductive Coercion in Romantic Relationships	2020	Fiji, specifically Suva. An ethnographic qualitative study using in-depth interviews and participant observation. The data were transcribed verbatim and analysed thematically using NVivo 10 and inductive analysis. 17 participants in the in-depth interviews. Young unmarried iTaukei (Indigenous Fijian) women, aged 18–26, attending university in Suva.	No relevant information	No relevant information	Describes family socialisation in gendered norms.
Mohd Ali, Fauzah Rahimah (Mohd Ali, 2025)	Unseen and Unheard: The Silent Suffering of Women in Sacred Contexts	2025	A case situated in a Malay-Muslim context in Malaysia. Qualitative case study/case report based on the lived experience of one patient: a middle-aged professional woman who became a second wife in a polygamous marriage.	It describes mechanisms of coercion and control in which the husband engages in narcissistic abuse.	No relevant information	Highlights the relevance to reproducing gender norms of socialisation in Malay-Muslim communities.
Rodgers, K.B.; Hust, S.J.T.; Li, J.; Kang, S.; Garcia, A.L. (Rodgers et al., 2023)	Sexual Scripts and Sexual Consent: Gender Stereotypes, Music-Media Messages, and Sexual Consent Expectancies Among College Men and Women	2023	United States, specifically a northwestern university (Washington State University). Quantitative study using an online survey. Participants viewed music videos with sexual/objectifying content and then completed measures on sexual stereotypes, experiences of sexual violence, perceptions of women in the videos, and sexual consent expectancies. The authors used linear mixed modeling with Maximum Likelihood. 364 participants. Undergraduate college students, aged 18–25, mostly White, recruited from general education communication courses.	No relevant information	No relevant information	Analyses the impact of sexual scripts of subjugating women in music.
Schneider, Nicole (Schneider, 2018)	The Mask of Happiness: Unmasking Coercive Control in Intimate Relationships	2018	USA (author) Conceptual/illustrative article. It is a professional discussion piece that defines and explains coercive control through a case vignette. The article includes a fictional case vignette (“Meredith and Ron”) used for illustration.	It describes the difficulties victims face in identifying the dynamics of psychological coercion in relationships.	This research identifies how the victim’s social circle fosters a culture of silence that makes it difficult for her to leave the abusive relationship.	No relevant information
Sudila Zilinska, Miroslava; Bianchi, Gabriel (Šudila Žilinská & Bianchi, 2025)	Beyond Yes and No: Discursive Constructions of Grey Area and Sexual Boundaries in Women’s Lives in Slovakia	2025	Slovakia. Qualitative study using feminist relational discourse analysis (FRDA). Data were collected through focus groups and serial individual interviews. The analysis combined Foucauldian discourse analysis and the Listening Guide approach. 30 participants. 18 women in 3 focus groups and 12 women in individual interviews (each interviewed twice). Adult women in Slovakia, aged 35–54, predominantly heterosexual, reflecting on their sexual experiences, boundaries, and grey-area situations.	No relevant information	No relevant information	Describes different sexual scripts of women.
Tullio, Valeria; Lanzarone, Antonietta; Scalici, Edoardo; Vella, Marco; Argo, Antonina; Zerbo, Stefania (Tullio et al., 2021)	Violence against women in heterosexual couples: A review of psychological and medico-legal considerations	2021	Italy (authors) Short narrative review. The authors conducted a comprehensive PubMed search focusing on intimate partner violence against women, attachment styles, risk factors, and the victim–perpetrator relationship.	It describes mechanisms of psychological coercion such as gaslighting, traumatic dependency, and power dynamics.	No relevant information	No relevant information
Vera Cruz, Germano Vera; Sheridan, Taylor (Vera Cruz & Sheridan, 2022)	The Normalization of Violence during Sex among Young Mozambicans Reportedly under the Influence of Pornography	2022	Mozambique, specifically urban areas around Maputo, Matola, Marracuene, and Boane. Mixed qualitative–quantitative study using semi-structured individual interviews. Interview responses were subjected to discourse thematic, semantic, and frequency analysis, with additional frequency comparison tests. 105 participants. Mozambican adolescents and young adults aged 16–22 who had already watched pornographic videos; 57 male and 48 female participants.	No relevant information	No relevant information	Describe sexual scripts that foster female subjugation.
Zapcic, Ian; Fabbri, Megan; Karandikar, Sharvari (Zapcic et al., 2024)	‘How Can I Love You if You Don’t Let Me Do this?’ Evaluating the Effects of the Red Pill Seduction Community Experienced by Intimate Partners	2024	Mainly the United States, with participants also from the United Kingdom and mainland Europe. Qualitative study using interpretative phenomenological analysis (IPA). Data were collected through six in-depth, semi-structured interviews, conducted by phone or Skype audio. 6 participants. Cisgender, heterosexual women who had been current or former intimate partners of men involved in The Red Pill (TRP) community. Participants were aged 20–38 at the time of the interview.	It describes mechanisms of psychological coercion such as control, confusion, and manipulation.	No relevant information	No relevant information

## Data Availability

No new data were created or analysed in this study.
